# Factors related to nurses’ knowledge and attitudes towards pain management: a cross-sectional study of 32 tertiary hospitals in Anhui province, China

**DOI:** 10.1136/bmjopen-2024-097514

**Published:** 2025-05-08

**Authors:** Jiadong Wang, Yidan Zhang, Shumin Bi, Shangui Chen, Zhaowen Peng, Zhiju Li, Chunxia Ren

**Affiliations:** 1 Anhui Medical University School of Nursing, Hefei, Anhui Province, China; 2 Department of Anaesthesiology, the First Affiliated Hospital of Anhui Medical University, Hefei, Anhui Province, China; 3 Department of Painology, the First Affiliated Hospital of Anhui Medical University, Hefei, Anhui Province, China; 4 Anhui Medical Information Institute, Hefei, Anhui Province, China; 5 Department of Nursing, the First Affiliated Hospital of Anhui Medical University, Hefei, Anhui Province, China

**Keywords:** Pain management, Knowledge, Nurses

## Abstract

**Abstract:**

**Objective:**

Recognising and managing pain was considered an essential clinical skill for nurses. This study aimed to assess nurses’ knowledge of pain management and the factors associated with it.

**Design:**

A cross-sectional survey was conducted in Anhui province, China, using the Knowledge and Attitudes Survey Regarding Pain (KASRP) (2014). Multiple linear regression analysis identified factors associated with nurses’ KASRP Scores.

**Setting:**

The study was carried out in 32 tertiary hospitals in Anhui province, China, from 21 May to 13 July 2023.

**Participants:**

6928 registered nurses.

**Outcome measures:**

The main outcome was the KASRP Score, reflecting nurses’ knowledge and attitudes towards pain management. We assessed the associations between sociodemographic factors, knowledge-sharing behaviours (KSBs) and KASRP Scores.

**Results:**

Among the 6928 nurses analysed, the average KASRP Score was 17.70±3.57, corresponding to a correct response rate of 44.55%. Gender (p=0.003), professional title (p<0.001), education level (p<0.001), hospital grade (p=0.002), work department (p<0.001), pain resource nurse status (p<0.001), receipt of pain training (p<0.001), level of concern for patient pain (p<0.001), and KSBS Score (p<0.001) significantly influenced knowledge and attitudes. XGBoost regression further identified pain training, clinical department and KSB as top predictors, with relative importance weights of 0.237, 0.170 and 0.135, respectively (R² = 0.268, MSE=38.3).

**Conclusions:**

This large-scale study revealed inadequate knowledge and attitudes regarding pain management among nurses in Anhui province, with outcome scores associated with various demographic and professional factors. These findings highlighted the need for continuous education, enhanced academic training and supportive institutional policies. Nursing leaders should implement targeted training initiatives and promote knowledge-sharing environments to strengthen pain management competencies among nurses.

**Strengths and limitations of this study**

- This study was one of the largest surveys of its kind, providing a comprehensive reflection of nurses’ level of pain management in the study region.
- The study thoroughly considered potential influencing factors on nurses’ knowledge and attitudes towards pain management and employed rigorous and appropriate statistical analyses.
- Strict quality control measures were implemented throughout the survey to ensure the authenticity and validity of the data.
- Due to time and funding constraints, the study was limited to tertiary hospitals and did not include lower-grade hospitals, which will be addressed in future research.

## Introduction

 Pain is an unpleasant sensory and emotional experience typically caused by, or resembling that caused by, actual or potential tissue injury, and serves as a manifestation of various underlying diseases.^1^ It is a key diagnostic feature for numerous conditions and an important indicator of disease severity, activity and prognosis, making it essential for evaluating medical and health services.^2^ A large prospective cohort study in Germany reported that 47.2% of patients experienced severe pain (Numerical Rating Scale Score ≥8) within 24 hours postsurgery, highlighting the need for nurses with extensive knowledge and skills in pain management.^3^

Clinical nurses played a pivotal role in making decisions and implementing strategies for pain management. Their knowledge and attitudes directly influenced whether patients’ pain symptoms were effectively addressed. Several studies^4 5^ indicated that nurses often had insufficient knowledge and demonstrated suboptimal attitudes towards pain management, which exacerbated patient suffering, delayed recovery and increased healthcare costs.

Most existing studies focused on single populations or small samples within individual centres.^6 7^ However, differences in region, society and culture may lead to variability in nurses’ pain management competencies.

The Knowledge and Attitudes Survey Regarding Pain (KASRP), developed by Ferrell and McCaffery in 1987 and revised in 2014,^8^ has been widely used to assess healthcare professionals’ pain-related knowledge and attitudes. This study assessed nurses’ knowledge and attitudes regarding pain management in tertiary hospitals in Anhui province, China, using the KASRP and the Knowledge Sharing Behaviour Scale (KSBS). By conducting a large-scale, multi-institutional survey, we aimed to provide nursing managers with evidence to develop more effective pain management strategies.

## Methods

### Study design

This multicentre study was conducted via the WeChat platform, using the Wenjuanxing platform to distribute the questionnaire. Prior to data collection, survey coordinators at each hospital received standardised training regarding the study’s objectives, as well as inclusion and exclusion criteria. The study adhered to principles of informed consent and voluntary participation. All participants provided informed consent, and personal information was kept strictly confidential. The survey took approximately 15–20 min to complete and could not be exited midway. Each mobile IP address was permitted only one submission.

### Participants

Participants were clinical nurses randomly selected from 16 cities in Anhui province through stratified cluster sampling. Two tertiary hospitals were chosen from each city, totalling 32 hospitals. From 21 May to 13 July 2023, nurses from tertiary general hospitals in the 16 prefecture-level cities of Anhui province participated in the survey.

Inclusion criteria were: (1) Possession of a valid nursing qualification certificate and active registration in clinical nursing, and (2) Informed consent and voluntary participation. Exclusion criteria were: (1) Non-salaried staff such as visiting nurses, (2) Trainee or resigned nurses, and (3) Nurses not engaged in clinical work during the study period (eg, those on leave or studying abroad).

Based on the literature,^9^ the estimated prevalence of low pain empathy among nurses was 54.4%. Using the stratified sampling formula $N=deff\frac{u^{2}p\left(1-p\right)}{\delta^{2}}$ where, p=0.544, *δ*=0.544×15%=0.0816, *deff*=1.5, u_0.05/2_=1.96, the required sample size per city was calculated as 213 participants. With two hospitals sampled in each of the 16 cities, the total sample size required was 213×16 × 2 = 6816. Accounting for a 10% non-response rate, the adjusted total sample size was 6816 × (1+0.10) = 7497.6, rounded to 7500 participants.

### Measurement tool

Sociodemographic data were collected using a customised general profile questionnaire and the 2014 version of the KASRP, along with the KSBS, to assess nurses’ knowledge and attitudes towards pain management. The general questionnaire, developed based on a literature review, included demographic variables such as gender, age, professional title, highest educational attainment, department, position and hospital grade. It also assessed pain management practices, including previous pain-related training, participation in a pain care team and the level of concern for patients’ pain. A pilot survey was conducted before formal data collection, and expert feedback was used to refine the questionnaire for clarity and completeness.

The KASRP (2014), developed by Ferrell *et al*,^10^ consists of 41 items: 22 true/false questions, 13 multiple-choice questions and 2 case analyses (each with two items). Each correct answer was scored as 1 point, and incorrect answers scored 0, yielding a total possible score of 41. Higher scores reflected greater knowledge of pain management. The scale demonstrated good internal consistency (Cronbach’s α>0.7) and test-retest reliability (r>0.8).^10^ A correct response rate of 80% was considered acceptable.

The KSBS, developed by Yi in 2009, has been widely applied in enterprises, schools and research teams.^11^ Chen^12^ translated and adapted it into Chinese, organising it into four dimensions: written contribution, organisational communication, personal interaction and community practice, comprising a total of 19 items. The KSBS used a 5-point Likert Scale ranging from 1 (‘none’) to 5 (‘always’), with a maximum score of 95. Higher scores indicated stronger knowledge-sharing behaviour (KSB). The Chinese version demonstrated high internal consistency, with a Cronbach’s α of 0.923.^12^

### Sampling frame

The sampling frame included all clinical nurses registered and employed in tertiary hospitals across 16 cities in Anhui province. According to data from the Anhui Provincial Health Commission (2022), there were approximately 221 000 registered nurses in the province. Based on healthcare workforce distribution and hospital bed data, an estimated 40%–50% (88 000–111 000) were employed in tertiary hospitals. Among these, approximately 48 000 nurses worked in the selected 32 tertiary hospitals. After excluding those in administrative, teaching or logistical roles, the final sampling frame consisted of around 43 000 clinical nurses.

### Random selection within hospitals

Within each hospital, participants were selected using stratified random sampling at the departmental level (eg, medical wards, surgical wards, ICU). Sample sizes were proportionally allocated based on the number of nurses in each department. Random selection was performed using computer-generated numbers via the Excel RAND function. The full staff list was obtained from the hospital’s human resources department, and random numbers were assigned to each nurse. Nurses were then selected according to departmental quotas. To maintain sampling accuracy (error <5%), a dynamic adjustment strategy was employed, including the use of a backup randomisation list.

### WeChat platform accessibility

Before launching the study, we confirmed with hospital nursing departments that WeChat was the primary communication platform in all participating hospitals, ensuring universal smartphone access among nurses. The electronic questionnaire was distributed via the ‘Wenjuanxing’ platform, compatible with both mobile and desktop versions of WeChat. Participants reviewed standardised instructions and, after confirming their understanding of the study’s purpose, completed the survey independently. To maximise accessibility and response rates, contingency plans were in place, including the provision of shared tablets during shifts and paper-based questionnaires when necessary.

### Handling non-response

Nurses who initially did not respond were reminded up to three times at 24-hour intervals. Persistent non-respondents were replaced with randomly selected nurses of similar rank from the same department to ensure the target sample quotas were met. Paper-based questionnaires were provided for nurses who encountered difficulties with the electronic format.

### Validation of the KASRP (2014) Scale in the study population

A pilot study involving 250 clinical nurses in Anhui province was conducted prior to the formal survey to evaluate the reliability and validity of the KASRP (2014) Scale. Internal consistency analysis yielded a Cronbach’s α coefficient of 0.891, indicating strong reliability. Construct validity was assessed using principal component analysis with varimax rotation. The results showed a Kaiser-Meyer-Olkin value of 0.840 and a statistically significant Bartlett’s test of sphericity (χ² = 1250.600, p<0.01), confirming good structural validity. These findings supported the use of the KASRP (2014) Scale to assess pain management knowledge and attitudes in this study population.

### Statistical analysis

Data entry was performed using Excel, and all analyses were conducted using SPSS V.26.0. Quantitative variables with normal distribution were expressed as mean±SD, while categorical variables were summarised as frequencies and percentages. Univariate analyses were performed using t-tests or analysis of variance. Pearson correlation was used to assess relationships between variables. Influencing factors were identified using multiple linear regression and XGBoost regression analysis. Two-tailed values of p<0.05 were considered statistically significant.

### Patient and public involvement

None.

## Results

Initially, 7530 questionnaires were distributed. Among these, 223 received no response and were subsequently replaced by randomly selected nurses of the same rank within the same department. In total, 7753 questionnaires were distributed, of which 7530 were returned and deemed valid. Questionnaires were excluded if submitted from repeated IP addresses or completed in under 5 min. A total of 602 questionnaires were excluded, yielding an effective response rate of 89.36%. Thus, 6928 nurses were included in the final analysis, comprising 282 men (4.1%) and 6646 women (95.9%).

Univariate analysis results for nurses with varying characteristics are presented in table 1. The analysis indicated that gender, age, professional title, education level, hospital grade, department, position title, role as a pain resource nurse, prior pain training, frequency of using pain assessment tools, and awareness of patients’ pain levels were all significantly associated with nurses’ knowledge and attitudes regarding pain management.

**Table 1:** Knowledge and Attitudes Survey Regarding Pain (KASRP) Scores and univariate analysis results for nurses with various characteristics (n=6928)

Characteristics		N (%）	Scores (mean±SD)	t/F value	*P value*
Gender	Male	282 (4.1)	16.77±3.24	−4.497	<0.001
	Female	6646 (95.9)	17.74±3.58		
Age (years)	20~30	2471 (35.7)	17.87±3.62	4.491	0.004
	31~40	3387 (48.9)	17.55±3.48		
	41~50	849 (12.3)	17.82±3.75		
	＞50	221 (3.2)	17.82±3.70		
Professional title	Nurse	790 (11.4)	17.14±3.50	19.006	<0.001
	Nurse practitioner	2556 (36.9)	17.79±3.59		
	Nurse-in-charge	3287 (47.4)	17.66±3.52		
	Associate professor of nursing or above	295 (4.3)	18.94±3.89		
Education status	College degree or below	986 (14.2)	17.06±3.57	20.952	<0.001
	Bachelor degree	5848 (84.4)	17.80±3.56		
	Master degree or above	94 (1.4)	18.61±3.37		
Years of clinical practice	<5	1512 (21.8)	17.78±3.53	2.384	0.067
	5~10	1738 (25.1)	17.83±3.60		
	10~15	2093 (30.2)	17.55±3.51		

The number and percentage of correct responses for each item on the KASRP Scale are shown in online supplemental table S1. The average correct response rate among the 6928 nurses was 44.55%. The highest correct response rate was observed for item 4 (92.44%), while the lowest was for item 38–2 (6.45%).

A correlation analysis between KASRP and KSBS Scores was conducted. Pearson correlation analysis revealed a statistically significant positive correlation (r=0.36, p<0.001), indicating that nurses with higher levels of knowledge and more positive attitudes towards pain management also demonstrated stronger KSB.

Multiple linear regression analysis was performed using the KASRP Score as the dependent variable. Independent variables were selected based on statistical significance (p<0.05) from univariate and correlation analyses. The variable assignment strategy is detailed in table 2, with unordered categorical variables converted into dummy variables. The analysis identified gender, professional title, education level, hospital grade, department, pain resource nurse status, pain training, concern for patient pain, and KSBS Score as significant predictors of nurses’ knowledge and attitudes. These factors explained 17.9% of the variance in KASRP Scores (table 3).

**Table 2:** Independent variable assignment instructions

Independent variable	Assignment method
Gender	Male=1, Female=2
Professional title	Nurse=1, Nurse practitioner=2, Nurse-in-charge=3, Associate professor of nursing or above=4
Education level	College degree or below=1, Bachelor’s degree=2, Master’s degree or above=3
Hospital grade	Tertiary A hospital=1, Tertiary B hospital=2, Tertiary unrated hospital=3
Working department	Outpatient and emergency=1, Oncology=2, Surgical=3, Medical department=4, Else=5
Whether they are pain resource nurses	No=1, Yes=2
Whether they have received pain training	No=1, Yes=2
Level of concern for patient pain	Very concerned=1, General concern=2, Not concerned=4
KSBS Score	Original values substituted into regression analysis

KSBS, Knowledge Sharing Behaviour Scale.

**Table 3:** Multiple linear regression analysis of influencing factors of Knowledge and Attitudes Survey Regarding Pain (KASRP) for nurses in Anhui province, China (n=6928)

Predictor	B	SE	Β	t	*Sig*
(Constant)	15.571	0.582	—	26.739	＜0.001
Gender	0.633	0.215	0.035	2.942	0.003
Professional title	0.439	0.083	0.092	5.316	<0.001
Education status	0.394	0.124	0.041	3.170	0.002
Hospital grade	−0.254	0.054	−0.056	−4.670	<0.001
Working department	−0.179	0.036	−0.058	−4.922	<0.001
Are you a pain resource nurse (refer: No)	0.647	0.139	0.056	4.668	<0.001
Have you received training on pain knowledge?	0.684	0.098	0.088	6.982	<0.001
Level of concern for patient pain	−0.473	0.098	−0.063	−4.844	<0.001
KSBS Score	−0.21	0.002	0.106	8.719	<0.001

KSBS, Knowledge Sharing Behaviour Scale.

**Figure 1:**
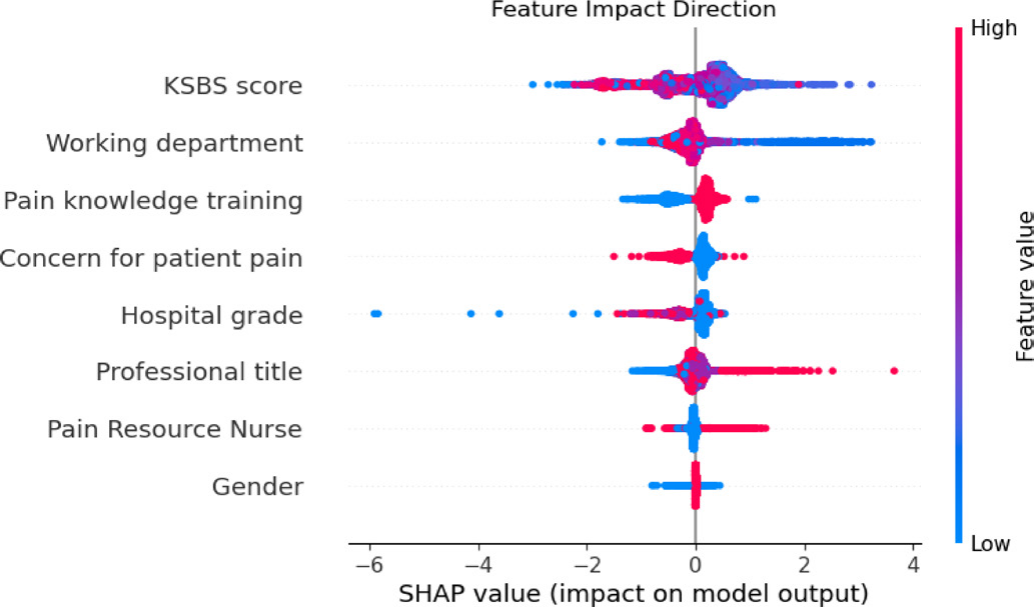
SHAP impact direction plot for XGBoost regression analysis. KSBS, Knowledge Sharing Behaviour Scale.

**Figure 2:**
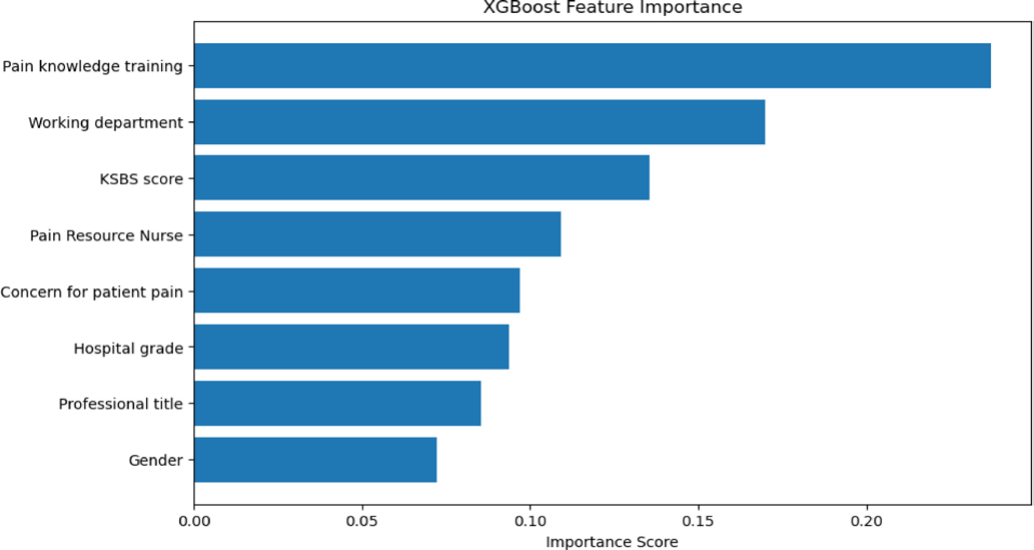
Feature importance plot for XGBoost regression analysis. KSBS, Knowledge Sharing Behaviour Scale.

The XGBoost model produced an R² of 0.268 with a mean squared error (MSE) of 38.3 (table 4). According to the XGBoost regression, pain training, department and KSBS Score were the top predictors, with relative importance weights of 0.237, 0.170 and 0.135, respectively. The contributions of each predictor are illustrated in figure 1 (SHapley Additive exPlanations (SHAP impact direction plot) and figure 2 (feature importance plot).

**Table 4:** Relative importance and standardised coefficients of variables in XGBoost regression analysis

Variable	Relative importance weight	Importance ranking	Standardised coefficient (β)	95% CI
Pain knowledge training	0.237	1	0.412	0.38 to 0.44
Working department	0.170	2	0.293	0.25 to 0.33
KSBS Score	0.135	3	0.181	0.15 to 0.21
Pain resource nurse	0.109	4	0.123	0.09 to 0.15
Concern for patient pain	0.097	5	0.095	0.06 to 0.12
Hospital grade	0.094	6	0.072	0.05 to 0.09
Professional title	0.085	7	0.044	−0.01 to 0.07
Gender	0.072	8	0.036	−0.02 to 0.05

KSBS, Knowledge Sharing Behaviour Scale.

## Discussion

We conducted a large-scale survey to assess nurses’ knowledge and attitudes towards pain management in Anhui province, China. Using the KASRP, a widely recognised tool for evaluating pain management knowledge and attitudes among nurses and nursing students,^4 13^ we found an average KASRP Score of 17.70±3.57, corresponding to an accuracy rate of 44.55%, which fell below the acceptable threshold established by the scale’s developers.

Our findings were consistent with those of Li *et al*,^14^ who investigated knowledge and attitudes towards cancer pain management among Chinese oncology nurses. However, our scores were significantly lower than those reported by Admass *et al*,^15^ who assessed pain knowledge among 138 pain resource nurses. This discrepancy may be attributed to the specialised training pain resource nurses typically receive.

The KASRP, developed by Ferrell and McCaffery, is a validated and widely used instrument for assessing healthcare professionals’ pain-related knowledge and attitudes.^6^ Globally, it has been instrumental in identifying educational gaps and guiding improvements in clinical pain management practices. The KASRP has been used in various countries—including those in Asia, as well as Italy, Greece, Spain, Canada, and the USA—to facilitate comparative studies of pain knowledge.^16^,^19^ For instance, a recent study in Vietnam validated the original KASRP and proposed a revised Vietnamese version.^19^ In Spain, the KASRP was effective in identifying low knowledge levels among nurses, reinforcing its utility as an assessment tool.^16^

As shown in online supplemental table S1, questions 38–2, 17 and 39–2 had the lowest correct response rates, reflecting nurses’ weaker competencies in pain assessment. These item-specific deficits offer valuable guidance for developing professional training programmes. One contributing factor may be nurses' reliance on patients’ expressions and body language, rather than prioritising patients’ self-reports—the gold standard for pain assessment.^3^ This can lead to the underestimation of pain, ultimately compromising intervention accuracy.^20^

Additionally, underdeveloped pain care infrastructure in China, limited educational resources and the relatively late publication of expert guidelines may contribute to the observed knowledge gaps.^21^ For example, the Chinese version of the expert consensus on adult postoperative pain management was only published in 2010—nearly two decades after similar guidelines appeared in Western countries.^22^ Although it set goals for maximising pain relief and maintaining optimal function, it lacked detailed guidance on assessment methods. Furthermore, China lacks clear policies on leadership responsibility and the implementation of pain care practices, and its pain management systems remain underdeveloped.^23^

In contrast, questions 14, 22 and 21 had the highest correct response rates, two of which addressed opioid use and one the concept of equivalent analgesia. This suggests that nurses in Anhui province were more knowledgeable about opioid management, possibly due to enhanced training and stricter institutional regulation. A survey of analgesic procurement across 793 public hospitals in China from 2013 to 2018 revealed a rapid increase in the use of analgesics, especially non-steroidal anti-inflammatory drugs (NSAIDs) and opioids.^24^ This widespread use has likely motivated nurses to deepen their understanding of these drugs to meet clinical demands.

However, Chinese nurses still lack access to comprehensive, standardised guidelines on analgesic care. Existing training remains fragmented and lacks systematic structure. Thus, there is a pressing need for national experts to develop detailed guidelines and expert consensus documents to support nurses in delivering effective pain care. Strengthening the pain care management system, enhancing nurse training and improving clinical competence in pain management are essential next steps.

These findings demonstrated that various factors influenced nurses’ knowledge and attitudes towards pain management. Gender was associated with KASRP Scores, possibly because women tend to exhibit greater sensitivity to pain perception,^25^ experience more pain, and possess higher levels of empathy. Nurses’ professional titles, which reflect their clinical experience, also significantly affected their knowledge and attitudes, as more experienced nurses generally acquire greater expertise. These results aligned with the findings of Li *et al*.^14^

Hospital grade and clinical department were also found to impact KASRP Scores. Higher-grade hospitals typically possess better resources and greater access to pain management information. Departments with more frequent exposure to patients in pain offer nurses richer clinical experience. These findings were consistent with Xie *et al*^26^ but differed from those of Al-Atiyyat *et al*,^27^ likely due to differences in regional training systems and local conditions.

Educational attainment, designation as a pain resource nurse and participation in pain training were positively correlated with KASRP Scores. These factors enriched nurses’ knowledge bases and enhanced their competency in pain management, as supported by Bouya *et al*.^28^ Higher academic qualifications correlated with stronger professionalism, better understanding of patient conditions and improved ability to manage pain symptoms. Pain resource nurses, having received additional training, were better equipped in this domain. Moreover, nurses’ attentiveness to patients’ pain varied significantly. Greater attentiveness, indicative of stronger empathy,^29^ motivated proactive pain management behaviours and elevated competence. Given empathy’s critical role in pain assessment and intervention,^30^ strategies to enhance empathy—such as targeted training^31^ and emotional regulation interventions^32^—are essential.

The observed KASRP accuracy rate of 44.55% was similar to that reported in Saudi Arabia (44.09%), suggesting that deficits in pain management knowledge are a global issue rather than a localised one.^33^ In contrast, the Spanish study reported a higher average score of 58.89%,^18^ indicating comparatively stronger foundational knowledge, though knowledge gaps persisted. Variability in pain management competency across countries may stem from differences in educational curricula, clinical training and cultural attitudes towards pain and opioid use, which influence both clinical behaviours and prescribing practices. Importantly, studies from multiple regions have highlighted persistent deficiencies in medication-related pain management, even in high-scoring populations. These results underscore the need for standardised, evidence-based training programmes to address global disparities and improve pain management outcomes.

The explanatory power of the multiple linear regression model (R² = 17.9%) was limited, potentially raising concerns about its robustness. To explore whether a more advanced model would improve explanatory power, XGBoost regression was performed, yielding an R² of 0.268 and an MSE of 38.3. Although XGBoost outperformed linear regression slightly, the improvement was modest. Previous studies reported similar explanatory power—13% in a study by Yu *et al* ^4^and 18.5% in a North China study on nurses’ pain knowledge and empathy^29^—suggesting our findings are within an acceptable range given the behavioural complexity of the topic. The results confirmed that while machine learning models like XGBoost can enhance prediction marginally, they offer limited gains in interpretability. Therefore, multiple linear regression remains an appropriate analytical approach under the current study conditions.

The positive correlation between KASRP and KSBS Scores further warrants attention. This finding aligns with prior research showing that collaborative learning environments improve nursing competencies, particularly in areas requiring continuous professional development.^34^ Teams implementing structured mentorship, interdisciplinary discussions and digital knowledge-sharing platforms have demonstrated enhanced clinical decision-making capabilities. KSB fosters team cohesion, collective innovation and organisational performance.^35^ Fundamentally, it reflects values such as altruism, empathy, fairness, responsibility and mutual assistance.^36^

Nurses who actively engage in knowledge sharing often possess higher levels of altruism and empathy, which motivate them to strengthen their professional competencies to better serve patients. In addition to benefiting recipients, knowledge sharing offers intrinsic rewards for the sharer. Research across various organisational contexts confirms the positive impact of knowledge sharing on both individual performance and team performance.^37^ Therefore, promoting KSB among nurses—by enhancing self-efficacy and establishing equitable performance evaluation systems^38^—is critical to advancing pain management expertise. Healthcare institutions could benefit from integrating structured knowledge-sharing strategies, such as formal mentorship programmes, interdisciplinary pain case reviews and digital learning platforms, to comprehensively enhance nurses’ capabilities in pain management.

The use of the WeChat and Wenjuanxing platforms for survey distribution was appropriate given their widespread adoption within China’s healthcare and academic sectors. However, potential selection bias may have occurred due to the exclusion of nurses who do not frequently use these platforms. To mitigate this, hospital administrators and department heads were actively engaged in survey dissemination to ensure broad participation across diverse nursing groups.

Although restricting partial responses and limiting submissions to one per IP address helped maintain data integrity and prevent duplicate entries, it may have introduced response bias by excluding individuals unable to complete the survey in one sitting or those using shared devices. These limitations were acknowledged, and future studies should consider multimodal data collection strategies to enhance inclusivity and response rates.

This study had several limitations. First, due to time and funding constraints, not all potential influencing factors were considered. Second, the survey was limited to tertiary hospitals in Anhui province and excluded lower-level hospitals, limiting generalisability. Third, the reliance on digital platforms may have excluded nurses unfamiliar with or without access to these tools, introducing selection bias. Future research should adopt diversified survey methods, expand the study scope to include lower-tier hospitals and integrate qualitative approaches for a more comprehensive analysis.

## Conclusion

Item-level accuracy rates on the KASRP provided valuable insights for designing pain management content in professional development training. The overall level of nurses’ knowledge and attitudes towards pain management was low, indicating the need for urgent improvement. Multiple factors—including KSB—were significantly associated with nurses’ pain management competencies. These findings offer actionable guidance for policymakers and healthcare administrators seeking to implement effective strategies that enhance nurses’ capabilities in pain assessment and treatment.

## Supplementary material

10.1136/bmjopen-2024-097514 online supplemental table 1

## Data Availability

All data relevant to the study are included in the article or uploaded as supplementary information.
