# Supplementary material for: Factors related to nurses’ knowledge and attitudes towards pain management: a cross-sectional study of 32 tertiary hospitals in Anhui province, China
Source: BMJ Open. 2025 May 8;15(5):e097514. doi: 10.1136/bmjopen-2024-097514 (PMC12067804; doi:10.1136/bmjopen-2024-097514)
Supplement: online supplemental table 1 [file bmjopen-15-5-s001.pdf]

Table S1 The correct answer rate of each item of KASRP scale for nurses in Anhui Province, China (N=6928)

Question	Correct answer	
	n	%
1.Vital signs are always reliable indicators of the intensity of a patient's pain.	2758	39.81
2.Because their nervous system is underdeveloped, children under 2 years of age have decreased pain sensitivity and limited memory of painful experiences.	1428	20.61
3.Patients who can be distracted from pain usually do not have severe pain.	2399	34.63
4.Patients may sleep despite severe pain.	1404	20.27
5.Aspirin and other nonsteroidal anti-inflammatory agents are NOT effective analgesics for painful bone metastases.	2056	29.68
6. Respiratory depression rarely occurs in patients who have been receiving stable doses of opioids over a period of months.	2801	40.43
7. Combining analgesics that work by different mechanisms (e.g., combining an NSAID with an opioid) may result in better pain control with fewer side effects than using a single analgesic agent.	4838	69.83
8. The usual duration of analgesia of 1-2 mg morphine IV is 4-5 hours.	1276	18.42
9. Opioids should not be used in patients with a history of substance abuse.	1320	19.05
10. Elderly patients cannot tolerate opioids for pain relief.	2455	35.44

11. Patients should be encouraged to endure as much pain as possible before using an opioid.	4477	64.62
12. Children less than 11 years old cannot reliably report pain so clinicians should rely solely on the parent's assessment of the child's pain intensity.	4831	69.73
13. Patient's spiritual beliefs may lead them to think pain and suffering are necessary.	4935	71.23
14. After an initial dose of opioid analgesic is given, subsequent doses should be adjusted in accordance with the individual patient's response.	6404	92.44
15. Giving patients sterile water by injection (placebo) is a useful test to determine if the pain is real.	1319	19.04
16. Vicodin (hydrocodone 5 mg + acetaminophen 300 mg) PO is approximately equal to 5 - 10 mg of morphine PO.	4642	67.00
17. If the source of the patient's pain is unknown, opioids should not be used during the pain evaluation period, as this could mask the ability to correctly diagnose the cause of pain.	692	9.99
18. Anticonvulsant drugs such as gabapentin (Neurontin) produce optimal pain relief after a single dose.	2736	39.49
19. Benzodiazepines are not effective pain relievers and are rarely recommended as part of an analgesic regiment.	5196	75.00

20. Narcotic/opioid addiction is defined as a chronic neurobiologic disease, characterized by behaviors that include one or more of the following: impaired control over drug use, compulsive use, continued use despite harm, and craving.	5869	84.71
21. The term ‘equianalgesia’ means approximately equal analgesia and is used when referring to the doses of various analgesics that provide approximately the same amount of pain relief.	5978	86.29
22. Sedation assessment is recommended during opioid pain management because excessive sedation precedes opioid-induced respiratory depression.	6284	90.70
23.The recommended route of administration of opioid analgesics for patients with persistent cancer-related pain is	2707	39.07
24.The recommended route of administration of opioid analgesics for patients with brief, severe pain of sudden onset, such as trauma or postoperative pain is	2569	37.08
25.Which of the following analgesic medications is considered the drug of choice for the treatment of prolonged moderate to severe pain for cancer patients?	3185	45.97
26. A 30 mg dose of oral morphine is approximately equivalent to:	3601	51.98
27. Analgesics for post-operative pain should initially be given	3775	54.49

28. A patient with persistent cancer pain has been receiving daily opioid analgesics for 2 months. Yesterday the patient was receiving morphine 200 mg/hour intravenously. Today he has been receiving 250 mg/hour intravenously. The likelihood of the patient developing clinically significant respiratory depression in the absence of new comorbidity is	1270	18.33
29. The most likely reason a patient with pain would request increased doses of pain medication is	4415	63.73
30. Which of the following is useful for treatment of cancer pain?	3580	51.67
31. The most accurate judge of the intensity of the patient's pain is	3749	54.11
32. Which of the following describes the best approach for cultural considerations in caring for patients in pain:	4101	59.19
33. How likely is it that patients who develop pain already have an alcohol and/or drug abuse problem?	3433	49.55
34. The time to peak effect for morphine given IV is	4203	60.67
35. The time to peak effect for morphine given orally is<br>______a. 5 min.	2936	42.38
36. Following abrupt discontinuation of an opioid, physical dependence is manifested by the following:	985	14.22
37. Which statement is true regarding opioid induced respiratory depression:	1887	27.24

38. Patient A: Andrew is 25 years old and this is his first day following abdominal surgery. As you enter his room, he smiles at you and continues talking and joking with his visitor. Your assessment reveals the following information: BP = 120/80; HR = 80; R = 18; on a scale of 0 to 10 (0 = no pain/discomfort, 10 = worst pain/discomfort) he rates his pain as 8.<br>38-1. On the patient's record you must mark his pain on the scale below. Circle the number that represents your assessment of Andrew's pain.	1167	16.84
38-2. Your assessment, above, is made two hours after he received morphine 2 mg IV. Half hourly pain ratings following the injection ranged from 6 to 8 and he had no clinically significant respiratory depression, sedation, or other untoward side effects. He has identified 2/10 as an acceptable level of pain relief. His physician's order for analgesia is "morphine IV 1-3 mg q1h PRN pain relief." Check the action you will take at this time.	447	6.45

39. Patient B: Robert is 25 years old and this is his first day following abdominal surgery. As you enter his room, he is lying quietly in bed and grimaces as he turns in bed. Your assessment reveals the following information: BP = 120/80; HR = 80; R = 18; on a scale of 0 to 10 (0 = no pain/discomfort, 10 = worst pain/discomfort) he rates his pain as 8.<br>39-1. On the patient's record you must mark his pain on the scale below. Circle the number that represents your assessment of Robert's pain:	1651	23.83
39-2. Your assessment, above, is made two hours after he received morphine 2 mg IV. Half hourly pain ratings following the injection ranged from 6 to 8 and he had no clinically significant respiratory depression, sedation, or other untoward side effects. He has identified 2/10 as an acceptable level of pain relief. His physician's order for analgesia is "morphine IV 1-3 mg q1h PRN pain relief." Check the action you will take at this time:	796	11.49
