# Supplementary material for: Factors related to nurses’ knowledge and attitudes towards pain management: a cross-sectional study of 32 tertiary hospitals in Anhui province, China
Source: BMJ Open. 2025 May 8;15(5):e097514. doi: 10.1136/bmjopen-2024-097514 (PMC12067804; doi:10.1136/bmjopen-2024-097514)
Supplement: Supplementary file 2 [file bmjopen-2024-097514.reviewer_comments.pdf]

PEER REVIEW HISTORY

BMJ Open publishes all reviews undertaken for accepted manuscripts. Reviewers are asked to complete a checklist review form and are provided with free text boxes to elaborate on their assessment. These free text comments are reproduced below.

ARTICLE DETAILS

Title (Provisional)

Factors related to nurses' knowledge and attitudes toward pain management: a cross-sectional study of 32 tertiary hospitals in Anhui Province, China

Authors

Wang, Jiadong; Zhang, Yidan; Bi, Shumin; Chen, Shangui; Peng, Zhaowen; Li, Zhiju; Ren, Chunxia

---

VERSION 1 - REVIEW

**Reviewer**	**1**
**Name**	**Maestro-Gonzalez, Alba**
**Affiliation**	**Hospital Universitario Central de Asturias**
**Date**	**31-Jan-2025**
**COI**	**None**

First and foremost, I would like to express my appreciation for the opportunity to review your manuscript, which addresses a highly relevant topic in clinical practice and pain management. The knowledge and attitudes of nurses towards pain management are crucial factors in ensuring high-quality patient care and safety. Your study makes a significant contribution to understanding this issue by providing valuable data on the factors influencing nurses' preparedness for pain management in hospital settings. However, after conducting a detailed evaluation of the methodological, statistical, and conceptual aspects of the manuscript, I believe that substantial modifications are necessary before it can be considered for publication. Therefore, my recommendation is to request major revisions, with the aim of enhancing the validity and impact of the findings presented.

The study has been designed as a cross-sectional survey-based study targeting nurses in Anhui Province, China. The use of the Knowledge and Attitudes Survey Regarding Pain (KASRP) (2014) is an appropriate choice given its widespread application in assessing knowledge and attitudes towards pain management. However, it is important that the authors provide a more detailed discussion regarding the global relevance and applicability of this scale. The KASRP has been extensively used in multiple countries, including Italy,

Greece, Spain, Canada, and the United States, among others, allowing for comparisons between different nursing populations. Incorporating a review of previous studies that have utilised the KASRP in various international contexts and comparing the findings of this study with those from other countries would strengthen the manuscript's impact and better contextualise the level of knowledge and attitudes towards pain management in China within a global framework.

Regarding data collection, the use of the WeChat and Wenjuanxing platforms for survey distribution is understandable within the current digital landscape. However, it is not mentioned whether measures were taken to control for selection bias associated with this method, such as the potential exclusion of nurses who do not regularly use these platforms. Furthermore, while the restriction that the survey did not allow partial responses and that each IP address could only submit one response suggests appropriate quality control, it may also have introduced bias in the response rate.

In terms of the measurement instrument, the KASRP (2014) scale has been validated in various nursing populations. However, it would be advisable to include further details regarding its validity and reliability within the specific population of this study. Although the authors mention an overall reliability coefficient ($\alpha > 0.7$), conducting an internal consistency analysis specific to this sample and discussing whether certain items exhibited low discrimination or reliability would be beneficial.

From a statistical perspective, the analyses conducted are generally appropriate for the study's objectives. Student's t-tests and ANOVA were used to examine differences in knowledge and attitudes across various sociodemographic variables, while Pearson correlations and multiple linear regression were employed to determine predictive factors for nurses' pain management knowledge. However, the linear regression model shows limited explanatory power, with a variance explained of only 17.9%, suggesting that additional factors not considered in the study may be influencing the results. A deeper discussion of this limitation is recommended, along with an exploration of whether a more complex model, such as multinomial logistic regression or structural equation modelling, could provide a more robust analysis.

Regarding the study's findings, the results indicate that nurses' knowledge of pain management is insufficient, with an average correct response rate of 44.55%, which is well below the 80% threshold recommended for the KASRP scale. Given that the KASRP has been used in numerous international studies, it would be highly beneficial for the authors to compare their results with those obtained in other countries. Such a comparison would help

assess whether the knowledge and attitude deficiencies observed in this sample are consistent with findings from other contexts or whether significant differences exist due to cultural, educational, or institutional factors.

An interesting finding is the positive correlation between knowledge-sharing behaviours (KSBS) and pain management knowledge, suggesting that nurses who share information with colleagues tend to have higher levels of knowledge in this area. However, this aspect is not explored in depth in the discussion. It would be advisable for the authors to expand their analysis of this relationship and consider its practical implications, such as the development of institutional strategies to promote knowledge exchange within hospital settings.

In the discussion section, the authors successfully contextualise their findings within the theoretical framework and compare their results with previous studies. However, there are several areas where this section could be strengthened. The authors are encouraged to elaborate further on the clinical applicability of their findings, discussing concrete strategies for improving pain management training within healthcare institutions. Furthermore, it would be important to explore how these findings could be translated into hospital policy changes, such as the implementation of mandatory training programmes or the establishment of specialised pain management teams within hospitals.

Finally, the conclusion section effectively summarises the study's main findings, but it would benefit from a more specific discussion of the practical recommendations derived from the results. Instead of a general conclusion on the need to improve nurses' education, it would be more useful to provide concrete recommendations, such as integrating specific pain management modules into continuing nursing education or developing evidence-based clinical guidelines to standardise pain management practices in hospitals.

By implementing these revisions, the manuscript will attain a stronger level of scientific rigour and make a more substantial contribution to the field of pain management in nursing.

Yours sincerely,

**Reviewer**	**2**
**Name**	**Lema, Girmay Fitiwi**
**Affiliation**	**University of Gondar, Department of Anesthesia**
**Date**	**12-Feb-2025**
**COI**	**None**

Abstract is not written in flawless ,there is reputation of ideas ,the grammars is not well accurate

page 7 line 22-24 requiring references ......regarding the rate of low level of empathy

.

VERSION 1 - AUTHOR RESPONSE

Reviewer 1: Dr. Alba Maestro-Gonzalez

1. ***The global relevance of the KASRP (2014) scale should be discussed, including its usage in other countries.***

   Response: We have now expanded the introduction and discussion sections to highlight the global relevance of the KASRP scale, citing studies from various countries. In the introduction section, we added "The Knowledge and Attitudes Survey Regarding Pain (KASRP), developed by Ferrell and McCaffery in 1987 and revised in 2014[1], is a widely utilized instrument for assessing healthcare professionals' knowledge and attitudes toward pain management.".

   In the discussion section, we added "The KASRP, originally developed by Ferrell and McCaffery, is a widely utilized and validated instrument for evaluating healthcare professionals' knowledge and attitudes toward pain management[1]. Recognized globally, the KASRP has been instrumental in identifying gaps in pain education and informing strategies for improving clinical pain management practices. The scale has been employed in multiple countries, including Asian countries, Italy, Greece, Spain, Canada, and the United States, facilitating comparative analyses of pain management knowledge worldwide[2-5]. For example, a recent study in Vietnam demonstrated that the original KASRP was reliable and valid, while also proposing a revised Vietnamese version of KASRP[5]. In Spain, a study analyzing determinants of pain management knowledge among nurses found that the KASRP effectively identifies low levels of knowledge, reinforcing its utility as an assessment tool[4]." These comparisons provide a broader context for the method we used.

2. ***Clarify potential selection bias due to the use of WeChat and Wenjuanxing platforms for data collection.***

   Response: We acknowledge the potential selection bias and have now explicitly stated the limitations related to digital access disparities. Regarding data collection, the use of the WeChat and Wenjuanxing platforms for survey distribution is appropriate given their widespread use in China's healthcare and academic sectors. However, potential selection bias may have arisen from the exclusion of nurses who do not frequently use these platforms. To mitigate this, hospital administrators and department heads were actively involved in distributing the survey to ensure broad participation across different nursing groups. Additionally, while restricting partial responses and limiting submissions to one per IP address helped maintain data integrity and prevent duplicate entries, it may have introduced response bias by excluding individuals who were unable to complete the survey in one sitting or those using shared devices. These factors have been acknowledged as limitations, and future studies should consider multi-modal data collection approaches to improve inclusivity and response rates. We have added this in the discussion and limitation section.

3. ***Provide specific validity and reliability data for the KASRP (2014) scale in this study population.***

   Response: We appreciate the reviewer's insightful suggestion and have now included a detailed assessment of the reliability and validity of the KASRP (2014) scale in this study population. A pilot study was conducted with 250 nurses, and the following psychometric analyses were performed:

   The internal consistency analysis revealed a Cronbach's α coefficient of 0.891, indicating strong reliability. To evaluate the construct validity, principal component analysis with varimax rotation was employed. The results yielded a Kaiser-Meyer-Olkin (KMO) value of 0.840 and a statistically significant Bartlett's test of sphericity ($\chi^2 = 1250.600$, $p < 0.01$), confirming the structural validity of the scale.

   These additional analyses confirm that the KASRP (2014) scale is a reliable and valid tool for assessing pain management knowledge and attitudes among nurses in this study population. We have updated the Methods section to include these findings (under the

subtitle **Validation of the KASRP (2014) Scale in The Study Population**), and we thank the reviewer for the valuable suggestion that has helped strengthen our manuscript.

4. ***The linear regression model has limited explanatory power (R^2=17.9%). Consider a more complex model.***

   Response: We appreciate the reviewer's concerns regarding the explanatory power of our multiple linear regression model. In response, we have expanded our discussion to clarify why multinomial logistic regression was not appropriate for our study. Since the dependent variable (nurses' pain management knowledge and attitudes) is a continuous score rather than a categorical variable, using logistic regression would result in significant classification bias, as the division of participants into "qualified" and "unqualified" groups would create imbalanced sample sizes.

   To further evaluate the robustness of our model, we conducted an XGBoost regression analysis, a machine-learning method that can capture complex relationships between variables. While the XGBoost model increased the explanatory power slightly (R^2 = 0.268), the improvement was not substantial enough to justify replacing the original linear regression approach. Previous studies have reported similar explanatory power (e.g., 13% in a study by Yu et al. [6] and 18.5% in a North China study on nurses' pain knowledge and empathy[7]), reinforcing that our results are within an acceptable range given the complexity of human behavior in pain management practices.

   Based on these findings, we maintain that multiple linear regression remains a valid and appropriate choice for this study. The additional XGBoost analysis supports our results and provides further validation of the factors influencing nurses' pain management knowledge and attitudes. We have updated our manuscript (methods, results, and discussion section) to reflect these additional analyses and appreciate the reviewer's insightful feedback.

5. ***Compare KASRP results with international findings.***

   Response: We thank the reviewer for this insightful response. The findings of this study reveal a 44.55% correct response rate on the KASRP scale, which is comparable to Saudi Arabia (44.09%), indicating that pain management knowledge deficits are a

global concern rather than an isolated issue[8]. In contrast, the average KASRP score in the Spanish study (58.89%)[4] was notably higher than the 44.55% correct response rate observed in this study, suggesting that nurses in Spain may have comparatively stronger foundational knowledge in pain management, though significant gaps remain. These variations in knowledge may be influenced by differences in educational curricula, clinical training, and cultural attitudes toward pain management. Additionally, cultural perceptions of pain and opioid use can shape both clinical attitudes and prescribing practices, further contributing to regional disparities in pain management competency. Still, various studies from around the world suggested that even in regions with higher overall scores, deficiencies in medication-related pain management remain a concern. These findings emphasize the need for standardized, evidence-based training programs to bridge knowledge gaps and improve pain management outcomes globally. We have added this in the discussion section.

6. ***Further explore the relationship between knowledge-sharing behavior (KSBS) and pain management knowledge.***

   Response: We appreciate the reviewer's insightful suggestion and have now expanded the Discussion section to explore the relationship between Knowledge-Sharing Behaviors (KSBS) and pain management knowledge in greater depth. The multiple linear regression analysis revealed a positive correlation between KSBS scores and KASRP scores, which merits attention. This aligns with previous studies demonstrating that collaborative learning environments enhance nursing competencies, particularly in areas requiring continuous professional development[9]. Groups that implement structured mentorship programs, interdisciplinary discussions, and digital knowledge-sharing platforms have reported improved clinical decision-making. Given these findings, healthcare institutions could benefit from integrating structured knowledge-sharing strategies—such as formal mentorship programs, interdisciplinary pain management case discussions, and digital learning platforms—to enhance nurses' competencies in pain management. We have added this in the discussion section.

7. ***Provide concrete recommendations for improving pain management training.***

Response: We appreciate the reviewer's valuable suggestion to strengthen the clinical applicability and policy implications of our findings. The results of this study highlighted critical gaps in nurses' pain management knowledge, emphasizing the need for structured training programs and hospital policy changes. Hospitals should integrate mandatory pain management modules into continuing education, incorporating case-based learning and simulation training to enhance clinical application. Additionally, the establishment of specialized pain management teams can facilitate collaborative decision-making and adherence to guidelines. Standardized pain assessment protocols and documentation practices should also be implemented to ensure consistency in care. Future studies should evaluate the effectiveness of these interventions on nursing competencies and patient outcomes. We have added this in the discussion section.

Reviewer 2: Dr. Girmay Fitiwi Lema

1. ***The abstract contains grammatical errors and redundant ideas.***

   Response: We thank the reviewer for this helpful suggestion. regarding the abstract's clarity and conciseness. In response, we have refined the abstract to eliminate redundancy, enhance readability, and ensure grammatical accuracy. Key revisions include streamlining sentence structure, removing unnecessary phrases, and strengthening the conclusion to clearly convey the study's implications. We reduced the word count while maintaining essential details. These improvements ensure that the abstract effectively summarizes the study's key findings in a concise and impactful manner.

2. ***Page 7, line 22-24 requires references regarding the low level of pain empathy.***

   Response: We appreciate the reviewer's insightful comment and acknowledge our oversight in omitting the citation. We have added appropriate citation to support it.

References

1. Ferrell B, McCaffery M. Knowledge and attitudes survey regarding pain. City of Hope Pain & Palliative Care Resource Center 2014.

2. Tafas CA, Patiraki E, McDonald DD, Lemonidou C. Testing an instrument measuring Greek nurses' knowledge and attitudes regarding pain. Cancer Nurs 2002;25(1):8-14. DOI: 10.1097/00002820-200202000-00003.
3. Moceri JT, Drevdahl DJ. Nurses' knowledge and attitudes toward pain in the emergency department. J Emerg Nurs 2014;40(1):6-12. DOI: 10.1016/j.jen.2012.04.014.
4. Maestro-Gonzalez A, Mosteiro-Diaz MP, Fernandez-Garrido J, Zuazua-Rico D. Determinants of Knowledge of Pain among Nurses in a Tertiary Hospital in Spain. Pain Manag Nurs 2021;22(3):394-401. DOI: 10.1016/j.pmn.2020.09.002.
5. Truong PV, An PTN, Huy TQ. Reliability and validity of revised instrument on pain management and its relationship between knowledge and attitudes among nurses in Vietnam. Bangladesh Journal of Medical Science 2024;23(1):160-170. DOI: 10.3329/bjms.v23i1.70710.
6. Yu W, Li D, Lu Y, Yang H, Ma X. Knowledge and Attitudes of Chinese Oncology Nurses Regarding Cancer Pain Management-a Cross-Sectional Study. J Cancer Educ 2021;36(5):1115-1123. DOI: 10.1007/s13187-020-01743-z.
7. Wu L, Chen X, Jia S, et al. Evaluating the relationship between pain empathy, knowledge and attitudes among nurses in North China: a cross-sectional study. BMC Nurs 2023;22(1):411. DOI: 10.1186/s12912-023-01577-2.
8. Al-Sayaghi KM, Fadlalmola HA, Aljohani WA, et al. Nurses' Knowledge and Attitudes Regarding Pain Assessment and Management in Saudi Arabia. Healthcare (Basel) 2022;10(3). DOI: 10.3390/healthcare10030528.
9. Magi L, Uibu E, Moi AL, et al. Collaborative learning linking nursing practice and education - Interview study with master's students and teachers. Nurse Educ Today 2024;139:106261. DOI: 10.1016/j.nedt.2024.106261.

VERSION 2 - REVIEW

**Reviewer**	**1**
**Name**	**Maestro-Gonzalez, Alba**
**Affiliation**	**Hospital Universitario Central de Asturias**
**Date**	**07-Apr-2025**
**COI**	

Dear Editor,

Given that the changes introduced have adequately addressed the weaknesses initially identified, and that the manuscript meets the scientific and editorial standards of the journal, my recommendation is to accept the article for publication without the need for further modifications.

I remain available for any additional clarification that may be required during the editorial process.

Sincerely,
